# Exosomes as a Nanodelivery System: a Key to the Future of Neuromedicine?

**DOI:** 10.1007/s12035-014-9054-5

**Published:** 2014-12-15

**Authors:** Arian Aryani, Bernd Denecke

**Affiliations:** Interdisciplinary Center for Clinical Research Aachen (IZKF Aachen), RWTH Aachen University, Pauwelsstrasse 30, 52074 Aachen, Germany

**Keywords:** Artificial exosome, Neurodegenerative disease, Extracellular vesicles, Multiple sclerosis, Diagnosis, MicroRNA

## Abstract

Since the beginning of the last decade, exosomes have been of increased interest in the science community. Exosomes represent a new kind of long distance transfer of biological molecules among cells. This review provides a comprehensive overview about the construction of exosomes, their targeting and their fusion mechanisms to the recipient cells. Complementarily, the current state of research regarding the cargo of exosomes is discussed. A particular focus was placed on the role of exosomes in the central nervous system. An increasing number of physiological processes in the brain could be associated with exosomes. In this context, it is becoming more apparent that exosomes are involved in several neurological and specifically neurodegenerative diseases. The treatment of these kinds of diseases is often difficult not least because of the blood-brain barrier. Exosomes are very stable, can pass the blood-brain barrier and, therefore, reveal bright perspectives towards diagnosis and therapeutic treatments. A prerequisite for clinical applications is a standardised approach. Features necessary for a standardised diagnosis using exosomes are discussed. In therapeutic terms, exosomes represent a promising drug delivery system able to pass the blood-brain barrier. One option to overcome the disadvantages potentially associated with the use of endogenous exosomes is the design of artificial exosomes. The artificial exosomes with a clearly defined therapeutic active cargo and surface marker ensuring the specific targeting to the recipient cells is proposed as a promising approach.

## Introduction to Small Vesicles

Over the last two decades, the intercellular communication became an intriguing research topic not solely in basic science but also in applied research. Besides short distance intercellular communication systems such as gap junctions and ligand-receptor interactions (e.g. cytokines), there do exist also long distance intercellular communication systems. These are either based on single molecules, like hormones, or on specific membrane-based structures, which can contain multiple molecules. One example for such a membrane-based structure is tunnelling nanotubes (TNTs), which are thin (50–700 nm) and up to 100 μm long actin containing tubes formed from the plasma membrane [1]. The TNTs can connect different kinds of cells and carry components of the cytoplasm between these cells such as vesicles and organelles [2]. TNTs may be involved in cell-to-cell communication [3], transfer of nucleic acids [2] and the spread of pathogens or toxins such as HIV and prions [4, 5]. Another example of membrane-based structures used for long distance intercellular communication is small vesicles. Small vesicles can mediate a cell-to-cell communication over long distances. They are suggested to control fundamental cellular responses such as intercellular signalling and immune reactions [6]. This is supported by the facts that small vesicles are relatively stable structures and the content of the vesicles is protected from degradation processes. For these reasons, among clinicians, the usage of small vesicles, namely exosomes, is widely discussed as a diagnostic tool and/or a potentially well-reputed therapeutic solution towards lot of diseases such as cancer or neurodegenerative diseases.

From the early stages of extracellular vesicle (EV) research, there was no clear definition regarding the identification and origin of small vesicles. It is unclear in some of the past studies what kind of vesicles they have applied for the experiments. This ambiguity is due to the technical limitations in the detection of such small-sized vesicles, the lack of a clear definition of each class and the impure isolates. Subsequently, it is not clear to which class of vesicles the presented data are correlated [7]. In the last few years, there was a major attempt to classify and identify these EVs and their cargo. Meanwhile, some general databases such as *Exocarta* (http://www.exocarta.org/) and *Vesiclepedia* (http://microvesicles.org/) are established to document data internationally and share this data for further investigations. However, improved methods are needed to isolate pure classes of extracellular vesicles. Also an international agreement on naming these vesicles can unify obtained information and help to enlighten on their biological function. Consequently, these improvements will facilitate scientists and clinicians to apply these vesicles for diagnoses or disease treatments.

In this review, we discuss the known characteristics of exosomes and try to clarify the cargo packaging and vesicle targeting. Furthermore, we focus on their potential roles in the treatment of neurological diseases such as multiple sclerosis (MS).

**Fig. 1:**
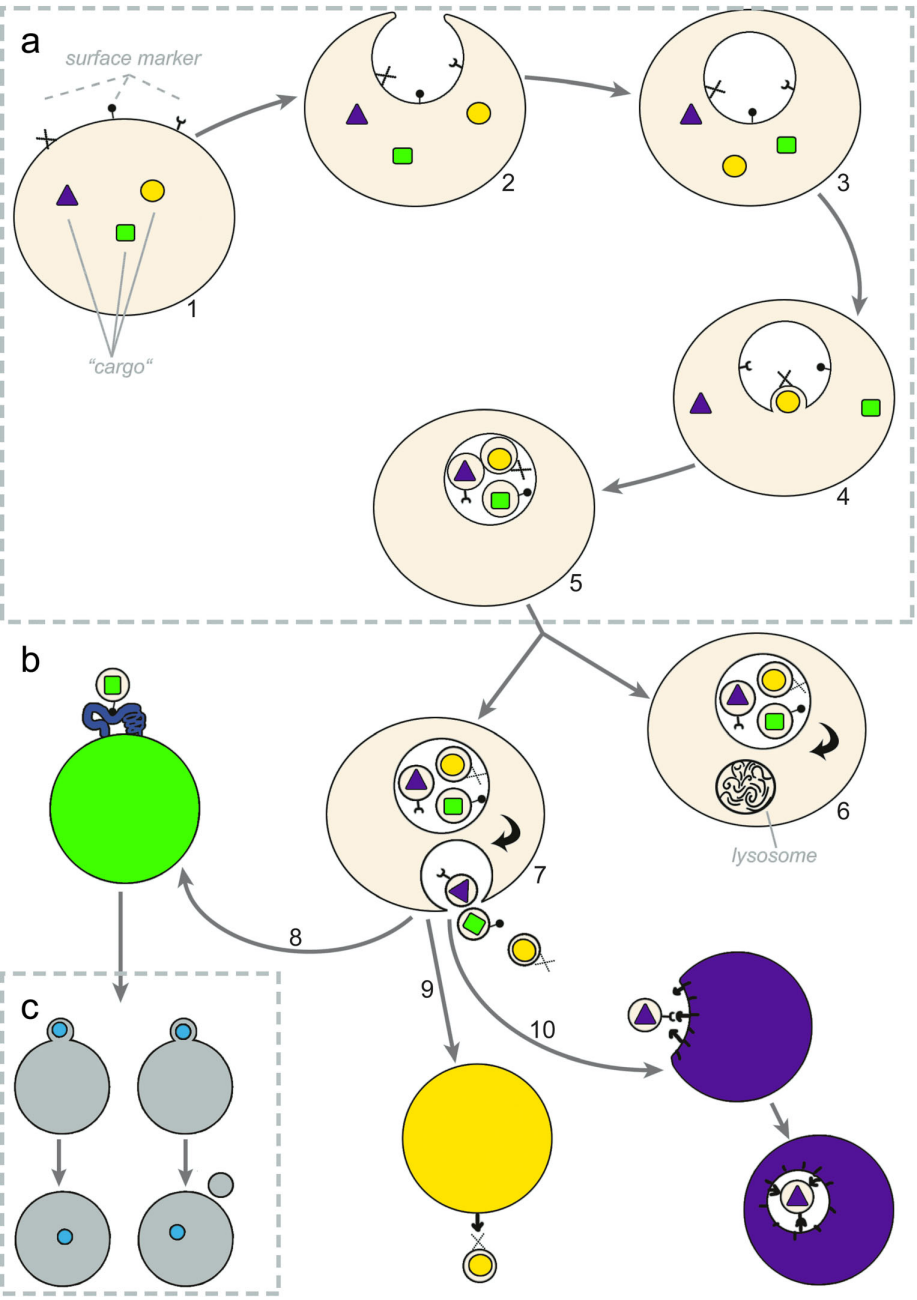
Formation and release of exosomes. **a** Exosome formation initiates by inward budding of plasma membrane to form MVBs. By formation of MVBs, receptors on the surface of plasma membrane locate inside the MVBs (*1*–*3*). Inward budding of MVBs results in the formation of internal vesicles inside the MVBs (*4*–*5*). By this stage, internal vesicles carry components from inside the cell such as cytosolic proteins or RNAs. Designated targeting molecules such as receptors are located on the outer surface of these vesicles similar to their location on the plasma membrane of the cell. **b** There are two possible destinations for the MVBs, which contain the internal vesicles. Either they are digested by lysosomes (*6*) or the MVB membrane fuses with the plasma membrane to release the internal vesicles now called exosomes (*7*). After reaching to their target cell, exosomes deliver their cargo either by adhesion via members of integrin family (*8*), via receptor ligand interaction (*9*) and/or via internalisation by endocytosis (*10*). **c** Exosomes deliver their cargo to the target cell either by fusion (*left part*) or hemifusion (*right part*). Fusion: the membrane of exosome and the target cell merge and result in an interconnected structure. Hemifusion: after releasing of cargo, the exosome membrane disconnected again from the plasma membrane of the target cell. *MVB* multivesicular body

EVs are small vesicles, which are secreted by prokaryotic and eukaryotic cells [8, 9]. Here, we distinguish between three classes of EVs, namely apoptotic bodies (ABs), microvesicles (MVs) and exosomes. It is known that most of the cells such as reticulocytes, dendritic cells (DCs), B cells, T cells, mast cells, platelets, epithelial cells, neurons, oligodendrocytes, Schwann cells and tumour cells are able to release exosomes [10–13]. However, the majority of exosomes detected circulating in vivo are platelet derived. The normal ratio of microvesicle found in blood plasma is nearly 80 % platelet derived, 10 % endothelial derived and 10 % leukocyte derived [11, 12]. Exosomes are present in physiological fluids such as plasma, lymph liquid, malignant pleural effusion, amniotic liquid, breast milk, semen, saliva and urine [14–18]. The conventional pathway of forming exosomes starts by inward budding of plasma membrane into forming multivesicular bodies (MVBs) (Fig. 1a 1–3) [19]. Alternatively, MVBs can be constituted from the trans-Golgi network [20]. Within these MVBs, exosomes can be formed by inward budding (Fig. 1a 4–5) and, subsequently, can be released by fusion of MVBs to the plasma membrane (Fig. 1b 7) [21]. It is proposed by van der Pol et al. that there is also a direct formation and release of exosomes from the plasma membrane. These vesicles also carry markers assigned to exosomes such as CD63 and CD81 and look identical to exosomes secreted by fusion of MVBs with the plasma membrane [19] (Table 1). In order to have a common understanding, in this review, we have defined ABs, MV and exosomes with the following characteristics:

- ABs are the result of apoptosis. They are heterogeneous in shape and contain materials such as DNA, RNA, histones and signalling molecules [19]. One signalling molecule for macrophages to remove ABs is the vitronectin receptor, which consists of two components (integrin α-V and integrin β-3 (CD61)) [22]. ABs originate from the plasma membrane, can be released from all cell types and are about 1–5 μm in size.
- MVs with the size of 20 nm–1 μm are formed due to blebbing with incorporation of cytosolic proteins. In contrast to ABs, the shape of MVs is homogenous. They originate from the plasma membrane and are observed in most cell types.
- Exosomes are vesicles with the size of 50–100 nm observed in most cell types similar to MVs with a difference: Instead of originating directly from the plasma membrane, they are generated by inward budding into MVBs. Fusion of these MVBs to the plasma membrane results in the release of the exosomes [19, 23].

**Table 1:** Vesicle formation

Extracellular vesicle	Observed by	Size	Origin	Formation due by
ABs	Apoptotic cells	1–5 μm	Plasma membrane	Apoptosis
MVs	Most of the cells	0.2–1 μm	Plasma membrane	Plasma membrane blebbing
Exosomes	Most of the cells	50–100 nm	MVBs	Inward budding

Although MVBs and MVs originate directly from the plasma membrane, the machineries involved in the formation and release of each are likely to differ [13]. Overall, MVs and exosomes are two separate classes of vesicles, which might overlap in size. In practical terms, they are indistinguishable from exosome. Therefore, MV in some studies is used as a general term for all small vesicles/particles which includes exosomes but not ABs [19]. Exosomes and MVs are generally smaller than ABs and, unlike them, contain rarely any DNA [11]. Most of the published data are from impure preparations of exosomes. The common protocol for exosome preparation is differential centrifugation to pellet the exosomes based on their size. However, by this method, the pellet includes also other vesicles in the size of exosomes such as MVs and large protein aggregates [7, 24]. It was observed that exosomes get released from most cells constantly; however, the level increases in the plasma of patients with inflammation, cell injury, thrombosis and platelet activation [11, 25, 26].

## Biogenesis and Targeting of Exosomes

Exosomes are formed by inward budding. Therefore, the orientation of proteins and lipids of the exosome membrane is equivalent to the plasma membrane of the parental cell (Fig. 1b 7) [27]. MVBs are late endosomes, which are refined by early endosome maturation. This maturation results in gradual changes in content and composition of the membrane [28]. The machinery involved in the formation of MVBs is relevant to exosome production. It is suggested for mammalian cells that phosphatidylinositol-3 kinase activity is required for both the formation of MVBs and the formation of exosomes (Fig. 1b 7) [27, 29]. Fernandez-Borja et al. showed that the inhibition of phosphatidylinositol-3 kinase results in swelling of several endocytic compartments and inhibition of MVB biogenesis [29].

There are two possible fates for the MVBs: Either they are designated to fuse with the plasma membrane (Fig. 1b 7), or alternatively, they fuse with a lysosome followed by digestion of the cargo (Fig. 1b 6) [13]. It was reported that cholesterol-rich MVBs are prone to release exosome and cholesterol-poor MVB are targeted to lysosomal digestion [30]. Another study showed that incorporation of membrane proteins such as growth factor receptors designates MVB to lysosomal degradation [27]. The molecular machinery involved in the biogenesis of exosomes can be dependent on or independent from endosomal-sorting complex responsible for transport (ESCART). The ESCART-dependent system is associated with accessory proteins such as programmed cell death 6 interacting protein (Pdcd6ip; also known as ALG2-interacting protein X (Alix)) and vacuolar protein sorting 4 (VPS4), which are used as exosome markers in many studies [13]. Alternatively, in the presence of sphingomyelinase, the exosome biogenesis might be independent from ESCART. Trajkovic et al. showed by inhibition of sphingomyelinase that the release of exosomes is significantly reduced [31]. Simons et al. showed that MVB formation can even be independent from ESCART and sphingomyelinase. By their results, tetraspanin proteins enriched in MVBs can play a major role in the formation of exosomes [32]. In addition, domains such as endosome-like domains, which are the site of endosomal protein binding in plasma membranes, serve also as a site for exosome biogenesis. These are the same sites wherefrom immunodeficiency virus (HIV) particles bud [33]; thereby, a number of cell-type-specific pathways bringing further insights into the classifications of EVs are reported [32, 34].

- Elements designating the fate of MVBs
  - Cholesterol content of MVBs
  - Membrane-bound proteins
  - ESCART machinery
  - Sphingomyelinase
  - Tetraspanin proteins

MVBs designated to exocytosis release the exosomes into the extracellular space by fusion with the plasma membrane. The release of exosomes can be constitutive or inducible depending on the cell type and the state of cell activation [17, 35]. For instance, immature DCs and epithelial cells release exosomes in a constitutive manner [36]. Another study has indicated that members of Rab family are involved in classical intracellular trafficking and in fusion of cellular compartment. It was also observed that a subset of this family, such as Rab27a and Rab27b, is involved in the secretion of exosomes [37]. For the last step of MVB fusion with the plasma membrane, it is also suggested that soluble *N*-ethylmaleimide-sensitive factor attachment receptor (SNARE) proteins play a crucial role [38].

It was observed that the protein content detected on the surface of exosome is significantly different from the ones on the plasma membrane. As an example, purified exosomes do not contain transferrin receptors (TfRs, a marker for plasma membrane and endosomes) [39]. The released exosomes are featured to reach their target cell to introduce the cargo. The targeting process is shown to be selective. Studies provided lines of evidence that although platelet-derived exosomes attach to both monocytes and neutrophils, transcription factors carried by these exosomes only get transferred to monocytes [40]. Another study showed that isolated B cell-derived exosomes specifically bind to follicular DCs [41]. It was demonstrated that the subsequent fusion is likely to be regulated by factors such as Ca^2+^ [42] and/or syntaxin-7 [43]. Although the efficiency of exosome transfer between cells is unknown, there are direct lines of evidence of exosome fusions to recipient cells. Using a fluorogenic dequenching assay with lipophilic dye R18-labelled exosomes, Montecalvo et al. have demonstrated that exosomes are capable of delivering their intraluminal cargo into the cytosol of the recipient cell [44]. The interactions of exosomes and target cells are target cell dependent and categorised as follows:

- Fusion to the target cell, such as adhesion via members of the integrin family or such as fusion of exosomes to monocyte with mediation of calcium and annexin V [41, 45, 46] (Fig. 1b 8).
- Receptor ligand interaction, such as antigen presentation and transfer of exosomes to B cells by mediation of complement receptor type 2 (Cr2 or CD21) [39, 45] (Fig. 1b 9).
- Internalisation by endocytosis, such as endocytosis of exosomes by DCs [10, 44, 47] (Fig. 1b 10).

It was observed that the efficiency of the fusion between exosome and recipient cell is also under control of environmental conditions as well as the state of the recipient cell such as maturation. As an example, fusion occurs more efficiently between exosomes and cells under acidic conditions [47, 48]. This consequently suggests a more frequent fusion within the tumour mass, which normally is more acidic, compared to the surrounding normal tissue. Additionally, Morelli et al. reported that the “exosome take-up ability” of BALB/c bone marrow–DCs decreases by the maturation of DCs, and consequently, immature DCs have a higher capacity to capture exosomes [49]. The fusion of exosomes to the target cell is, for at least a percentage of exosomes, not a complete fusion but just a hemifusion sufficient to release their content to the recipient cell (Fig. 1c) [44].

- Fusion of exosomes to the target cell
  - Via internalization by endocytosis
  - Via members of the integrin family
  - Via receptor ligand interaction

## Cargo of Exosomes

Initially, it was assumed that the content of exosomes is random due to the engulfed part of the cytoplasm packaged by the membrane blebbing [50]. Later, it was observed that the content of exosomes released by mesenchymal stromal cells (MSC) differs from their parental cells, probably caused by selective packaging. Although the sorting mechanisms of nucleic acids and proteins are poorly understood, there are some suggestions for sorting proteins inside exosomes, such as sorting via ESCART, via lipid and/or protein affinity or via sorting by protein incorporation into detergent-resistant protein complexes [17]. It is mentioned that exosomes contain some common and some cell-type-specific proteins [28]. Thery et al. reported that exosomes do not contain proteins that originated from the nucleus, mitochondrion, endoplasmic reticulum or Golgi apparatus. Instead, proteins identified in exosomes were also observed in the cytosol and plasma membrane [36]. In contrast, a subsequent study by Record et al. reported that exosomal proteins could also originate from the endocytotic compartment, Golgi and nucleus, but rarely from the endoplasmic reticulum or mitochondria [17].

RNAs detected in exosomes of MSCs consist of mainly messenger RNA (mRNA) and microRNA (miRNA). No track of 18S or 28S ribosomal RNA was detected. Indeed, in these exosomes, the portion of mRNAs is relatively small, while majority of small RNAs as well as miRNAs in precursor form were observed [51]. This observation suggests that the packaging of nucleic acid is not a random encasing of cytoplasmic content. Furthermore, there are essential differences between mRNA transcripts in parental cells compared to mRNA transcripts detected in exosomes, namely mRNA might be enriched in exosomes but not detectable in parental cells [10]. Such a selection was also observed for a number of detected miRNAs, which are assumed to be exclusively packed to exosomes [26]. Baglio et al. have observed that some miRNAs were present in both exosomes and parental cells. Nonetheless, some miRNAs appeared to be selectively sorted to exosomes and are not detectable in the parental cells. This observation supports the existence of control mechanisms for selective packaging of miRNAs for at least MSCs [26]. Likewise in AZ-P7a cells (a metastatic gastric cancer cell line), an enrichment of let-7 miRNA family members (tumour suppressor genes that target oncogenes) in exosomes was observed. The exosomal release of let-7 miRNAs into the extracellular environment maintained the oncogenesis and invasiveness of AZ-P7a cells by at least partially neutralising the inhibitory effects of let-7 miRNAs on their targeting oncogenes such as RAS and HMGA2 [10, 12, 52]. In another study, it was shown that the enrichment of miRNAs in the exosomes derived from DCs is selective as some miRNAs are detected in parental cells but not in the exosomes and vice versa [44].

As a matter of fact, miRNAs are delivered to distant cells by either miRNA containing exosomes or via free miRNA-molecules bound to Argonaute 2 (Ago2). It was shown that Ago2 is not only bound to free extracellular miRNAs but also to miRNAs within exosomes and other membrane-derived vesicles [12]. In most cases, miRNA was found single stranded in exosomes, yet precursor hairpin miRNAs were also detected [12, 51]. It is hitherto not clear if Ago2 is necessary for miRNA export [12].

The regulatory functions of miRNAs are accomplished through the RNA-induced silencing complex (RISC) [53]. It is believed that the mature miRNA not connected to RISC is not functional. Pre-miRNA can be loaded with RISC followed by cleavage into functionally mature RISC-loaded miRNA. The favoured secretion of pre-miRNA into exosomes suggests an important physiological role after being taken up by target cells and also supports the thesis that the content of exosomes is not just random [51].

Notwithstanding, miRNAs isolated from plasma exosomes, which are mostly platelet derived, has a significant different composition compared to platelets and peripheral blood mononuclear cells. As a consequence, many miRNAs are uniquely present in exosomes isolated from plasma [11]. The number and content of exosomes consistently vary based on the microenvironmental conditions of the cells and, particularly, if cells are subjected to stress factors [26]. It is shown that the miRNA content of exosomes in the plasma is different between normal and tumour-induced tissues [11]. Breast cancer cells produce exosomes with a changed pattern of miRNA [54]. Another example is the stimulated release of exosomes by increase of intracellular Ca^2+^ in neutrophils [38, 55]. Such stimulations also affect the content of exosomes. Finally, the miRNA content of exosomes also depends on the maturation state of the parental cell. The miRNA content of exosomes derived from mature DCs, which promote immunity, and the miRNA content of exosomes derived from immature DCs, which downregulate T cell responses, were observed to be significantly different. However, there is no significant difference in the amount of miRNA between exosomes derived from mature DC and immature DC [44]. It is important to mention that ABs, MVs and exosomes contain fundamentally different RNA profiles. For instance, MVs isolated from cell culture often do not contain a considerable amount of RNA. Ribosomal RNA is primarily found in ABs [56]. A selective loading is not only observed for protein and RNA but also for other types of molecules packed into exosomes. Regarding DNA as a content of exosomes, there exists a disagreement. One study announced that exosomes contain no DNA [10]; later, it was observed that astrocyte-derived exosomes might contain mitochondrial DNA [57, 58]. This discrepancy in the reported data demonstrates the need of further investigations in this field.

To sum up, exosomes play an important role in long distance cell-cell communication. They mainly contain a selection of different physiologically active proteins, miRNAs and mRNAs. Based on the parental cell status and/or the microenvironmental conditions, the content and the number of exosomes are varying.

## Detection of Exosomes

Although exosomes originated from a parental cell, their composition is to some extent different from the parental cell as mentioned above. Many plasma membrane proteins are depleted, and therefore, exosomes are distinguishable from shed plasma membranes [28]. Nevertheless, there are a number of proposed exosome reference markers such as lysosomal-associated membrane protein 2 (Lamp-2) and Rab 5B, which is a member of the RAS oncogene family [47]. Also, proteins such as Pdcd6ip, tumour susceptibility gene 101 (TSG101), tetraspanin proteins resulting from exosome formation in MVBs (CD9, CD63, CD81 and CD82) and proteins enabling intracellular membrane fusion and transportation [36] are often used as markers to detect and identify exosomes [23]. There are kits to detect exosomes in vivo available such as Exosomal Cyto-Tracer (The lentivector based Cyto-Tracer (Biocat GmbH) expresses the tetraspanin CD63, CD9 or CD8, which are fused to GFP or RFP. In this way, exosomes are marked and long-term and in-depth experimentation is enabled). However, these proposed reference markers are also present in MVs and ABs [56]. Therefore, they are not sufficient to establish a method to isolate/detect a pure population of exosomes. As a consequence, there is a need of improved techniques to detect exosomes in vivo and in vitro in a more specific manner.

## Exosomes and the Central Nervous System

Exosomes represent a major step in the diagnosis and treatment of many neurological diseases, such as Alzheimer’s disease (AD) and MS. In this context, the ability of exosomes to cross the blood-brain barrier (BBB) [59] is important in two aspects: First, considering the central nervous system (CNS) is highly protected and consequently not easily accessible, information regarding the cells in the CNS can be obtained via exosomes that passed through the BBB. Second, exosomes not only cross the BBB from the brain towards the blood and vice versa, but also can target a specific cell type and deliver the protein or nucleic acid content into their target cell [60]. Therefore, an exosomal delivery system potentially can transport pharmaceutically active substances and genetic materials across the BBB into the CNS. In order to use this kind of delivery system more efficiently, it is beneficial to know the cell-specific functions of exosomes as well as their biological roles in the brain. It is indicated that exosomes are linked to a number of different biological processes in the CNS, such as their crucial role in synaptic plasticity, regulation of myelin membrane biogenesis, as well as transfer of proteins or nucleic acid locally to highly polarised structures like neurons [6].

**Fig. 2:**
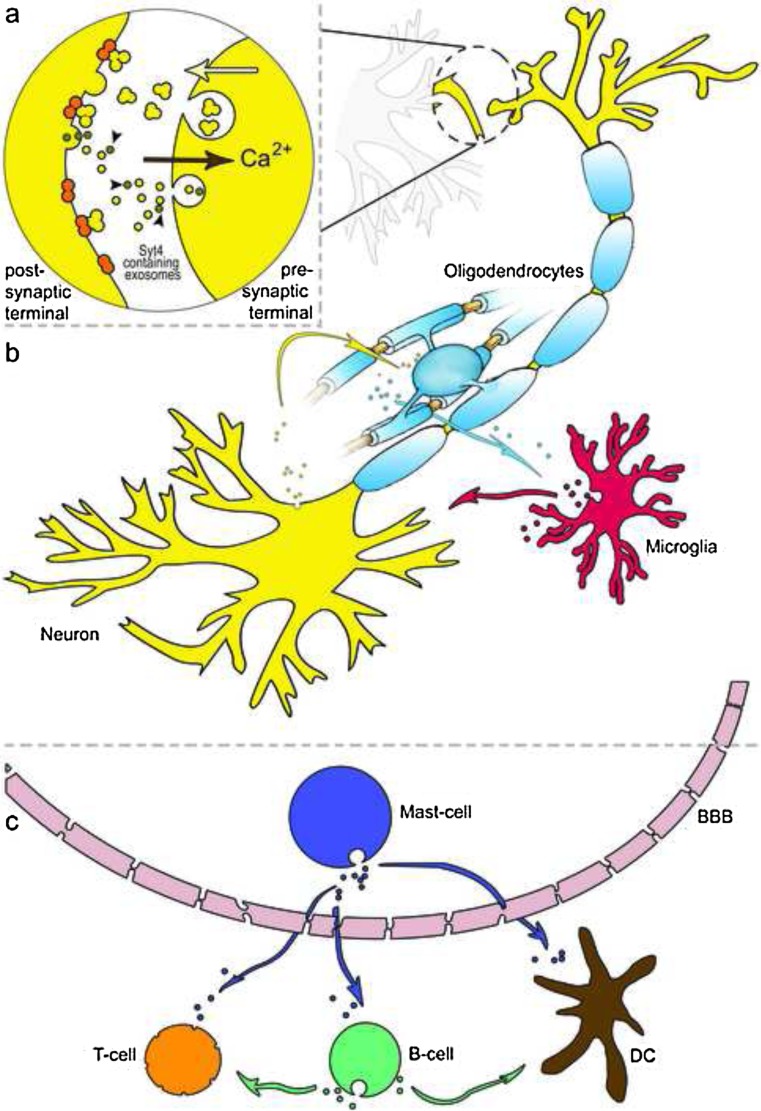
Active role of exosomes in the central nervous system. **a** Exosomes play a crucial role in synaptic plasticity. Syt4 is a membrane-trafficking protein and is essential for retrograde signalling. It was observed that exosome containing Syt4 (demonstrated by *brown circles*, indicated with *dark arrows heads*) are released from presynaptic terminal and thereby the entire pool of Syt4 in postsynaptic terminal is provided by exosomes from the presynaptic terminal. The *yellow arrow* presents the release of neurotransmitters (yellow triple-circle shapes) from presynaptic terminal and binding of them to neurotransmitter receptors (*orange two-circle shapes*). Release of neurotransmitters allows entry of Ca^2+^ to the presynaptic terminal and activates fusion of exosome-containing MVBs to the plasma membrane. **b** In the CNS, different cell types such as neurons, oligodendrocytes and microglia release exosomes (exosomes of each cell type are performed by *small circles* coloured in parental cell colour). Released exosomes reach different cell type in distance and deliver their cargo to other cells. Therefore, cells in the CNS can communicate with each other and help to regulate their function. **c** Exosomes derived from resident cells in the CNS can pass through the BBB. Mast cell-derived exosomes (*dark blue circles*) can pass through the BBB and are able to activate B and T cells or induces DCs to become efficient antigen-presenting cells. B cell-derived exosomes, in turn, can stimulate T cells or transfer MHC class II proteins to the surface of follicular DCs, which do not express these molecules

Among other things, it is proposed that exosomes play a role in synaptic plasticity. This could be achieved by the delivery of specific sets of proteins, mRNAs and miRNAs from the postsynaptic to the presynaptic terminal via exosomes [61]. Also the transport from presynaptic to postsynaptic terminal via exosomes was observed for synaptotagmin 4 (Syt4, a membrane-trafficking protein). Korkut et al. have shown that the entire pool of postsynaptic Syt4 is provided from presynaptic exosomes. Considering Syt4 is essential for retrograde signalling, the Syt4 exosomal transport supports the presynaptic control of retrograde signals [62]. The role of exosomes in synaptic plasticity is also supported by the detection of MVBs in neurons [63] and the fact that the MVB fusion to plasma membrane is linked to synaptic activity [64] (Fig. 2a).

The regulatory function of exosomes involved in myelin membrane biogenesis was observed between glial cells and axons (Fig. 2b). It is described that exosomes on the one hand contribute to eliminate overproduced myelin membranes [65] and, on the other hand, participate in myelin membrane biogenesis [66].

As a matter of fact, most if not all of the cell types in the CNS release exosomes. The exosome cargos in the CNS vary based on the cell of origin as well as the cells’ health/stress/disease state and can be changed in response to environmental situation [67]. Consequently, in the CNS, exosomes are involved in different procedures such as communication and regulation. Their role in communication is supported by the facts that (i) exosomes are intercellular vehicles for RNA transfer within the CNS [10] and (ii) exosomes also contain abundant proteins involved in cell-cell communications such as heat shock proteins and tetraspanins [35, 61, 65].

## Exosomes Derived from Different Cell Types in the CNS

Here, we introduce several known cell-type-specific exosomes such as neuronal, glial (microglia and macroglia) and mast cell-derived exosomes. Neurons as well as most glial cell types release exosomes. Besides neuron-specific components, neuron-derived exosomes largely resemble non-neuron-derived ones [61] and mediate a regulatory as well as communicative function [68]. Neurotransmitters released from the pre- to postsynaptic terminal allow Ca^2+^ entry to the neuron through synaptic receptors (Fig. 2a). This activates MVB fusion to the plasma membrane followed by the release of exosomes. Thus, more active neurons within a circuit are able to release more exosomes than less active or immature neurons [69, 70]. Released exosomes are targeted to glial cells, other neurons or the same neuron as well [69]. On the one hand, the neuron-derived exosomes help, inter alia, to communicate among neurons and oligodendrocytes resulting in myelination (Fig. 2b). On the other hand, oligodendrocyte-derived exosomes are endocytosed by neurons. Taken together, neuronal activity influences the cargo transfer from oligodendrocyte-derived exosomes to neurons. This suggests that neuronal activity has a regulatory function regarding receiving glial-derived components via the exosomes [65, 67, 70, 71].

Besides its function as a pure molecule transmission system, microglia-derived (resident macrophages in the CNS [72]) exosomes contain an intact metabolic pathway with all the enzymes necessary for anaerobic glycolysis/lactate production. In this way, microglia-derived exosomes can deliver lactate to neurons, which support the energy balance during synaptic activity. It is shown that these exosomes also contain functionally active CD13. CD13 is able to catabolise neuropeptides (i.e. methionin- and leucine-enkephalin), preventing the enkephalin-mediated cAMP decrease (Fig. 2b) [73].

Oligodendrocytes are a subclass of macroglia and support neurons by producing neurotropic factors [74]. They are responsible for myelin forming, which includes an extremely high membrane synthesis capacity. To remove excess membrane, oligodendrocytes release part of the membrane in the form of exosomes. It is proposed that microglia take up these exosomes by macropinocytosis. This process directs the incoming cargo away from compartments with pro-inflammatory receptors. Consequently, the antigen-presenting function of microglia is infiltrated for the proteins taken up via the exosomes. Due to the constitutive macropinocytosis clearance of exosomes by (perhaps a specific subset of) microglia, these systems provide an immunologically silent clearance of oligodendroglia membrane and myelin proteins [75]. A balance in myelin synthesis is also ensured by the level of proteolipid protein (PLP). PLP can be packaged into exosomes whose release is regulated by cytosolic Ca^2+^ (Fig. 2b) [76].

Mast cells are effector cells of the innate immune system and resident in most tissues characteristically surrounding the blood vessels and nerves [77]. They are especially prominent in tissues in close contact with the outside environment such as the skin, mucosa of the lungs and the digestive tract. Like macrophages, they are resident in the brains of many species. They enter the CNS during development via penetrating the blood vessels with which they remain associated [78]. In the brain, almost 97 % of mast cells lie on the abluminal side of the blood vessels [79]. Like lymphoblasts, mast cells can move through the normal brain even in the absence of inflammation, with rapid entry mediated via the BBB passage [80]. Because brain mast cell-derived exosomes can, as mentioned, pass the BBB, they can present the CNS antigens to the immune system outside of the CNS. It is known that mast cell-derived exosomes activate B and T cells (Fig. 2c). Potentially, mast cell-derived exosomes also induce maturation and activation of DCs due to cross presentation of antigens to T cells [71]. It was also observed that exogenous antigens in mast cell-derived exosomes are both in naïve and in processed form [71]. Mast cell-derived exosomes often carry two heat shock proteins (hsp60 and hsp70), which are known for their adjuvant activity. Therefore, even in the absence of conventional adjuvants, they are highly efficient in inducing an antibody response in vivo.

## Exosomes Involved in Neurological Diseases

Besides the biological roles in the CNS outlined above, the appearance of specific exosomes correlate with a number of known neurological diseases such as Huntington’s disease (HD), Parkinson’s disease (PD), AD, amyelotrophic lateral sclerosis (ALS) and MS (Table 2). Various numbers of neurological diseases such as HD, PD, ALS and AD are associated with the spread of a specific misfolded protein via the exosome within the CNS [131].

**Table 2:** PubMed publications for selected neurological diseases (18th August 2014)

Search term	NLM	References	PMC
Exosome	2908		7675
Alzheimer/AD and exosome	20	[81–100]	432
Multiple sclerosis/MS and exosome	8	[101–108]	601
Parkinson/PD and exosome	8	[109–116]	223
Huntington/HD and exosome	4	[113, 117–119]	115
Traumatic brain injury and exosome	4	[120–123]	118
Neuroinflammation and exosome	2	[124, 125]	190
Multiple system atrophy and exosome	2	[126, 127]	62
Amyotrophic lateral sclerosis/ALS and exosome	4	[103, 128–130]	195

*NLM* National Library of Medicine, *PMC* PubMed Central

HD is caused by CAG trinucleotide repeat expansion in the huntingtin gene (Htt) leading to altered histone acetylation and formation of mutant Htt [132]. MVBs in neurons have been shown to contain Htt protein aggregates, and the pathology of HD is closely tied to the MVB function of recycling and releasing these proteins via the exosomes [64]. A therapeutic approach towards HD is the decrease of mutated Htt expression via small RNA carried and delivered by exosomes. Theoretically, developed exosomes carrying small RNA can pass the BBB and facilitate the inhibition of mutated Htt expression.

PD is one of the most common neurodegenerative diseases among people over 50 years old. PD is characterised by degeneration of dopaminergic neurons in the *substantia nigra pars compact* and the presence of Lewy bodies, primarily composed of fibrillar α-synuclein (α-Syn) and ubiquitinated proteins [133]. Exosomes released from injured neurons contain α-Syn and are involved in the progress of PD by transferring the α-Syn to other neurons [109]. It was also reported that α-Syn can induce the release of exosomes from microglia cells [134]. These MHC II and membrane-bound tumour necrosis factor-α (TNF-α) containing exosomes can induce apoptosis. Therefore, it is proposed that exosomes can be mediators of α-Syn-induced neurodegeneration in PD [134].

For AD, it has been reported that amyloid precursor protein (APP) cleavage to β-amyloid occurs in early endosomes followed by routing the β-amyloid to MVBs. In detail, Rajendran et al. have detected APP and the β-amyloid precursor protein-cleaving enzyme (BACE) in early endosomes. By immunogold labelling, they identified β-amyloid in MVBs, which suggests that the secretion of β-amyloid peptides can be associated with exosomes. In the brain sections of AD patients, consequently, exosomal proteins (Pdcd6ip and flotillin-1) were accumulated in plaques, whereas they were almost absent in the brain sections of the control group. Based on these data, it is proposed that exosomes could provide a conducive environment for amyloidogenic fibril formation, and subsequently, exosomes play a role in the pathogenesis of AD [6, 135, 136].

ALS is a neurological motor neuron disease in which specific motor neurons in the brainstem, cortex and spinal cord are degenerated. Although the mechanisms involved are largely still unknown, in recent years, it has been shown that ALS is a non-cell-autonomous disease that implies the interaction between motor neurons and glial cells. In a mouse model, Basso et al. [137] have shown that on the one hand the overexpression of a mutated copper-zinc superoxide dismutase 1 (SOD1) in primary astrocytes is linked to a general reduction of total amount of secreted proteins. On the other hand, the group could observe an increase in the level of a selected number of proteins known to be released by exosomes [138]. Both, the cultivation together with astrocytes expressing the mutated SOD1 and the cultivation together with exosomes derived from these transgenic astrocytes, were sufficient to induce motor neuron death in vitro. A concentration dependency could be shown by the fact that the motor neuron viability decreased with increased concentrations of the exosomes, which instead did not affect the neuron viability. In contrast, exosome preparations from astrocytes expressing the unmutated SOD1 did not have an influence on the motor neuron viability although the mutated/unmutated SOD1 was taken up by the motor neuron cells in both cases. At least in the mutant SOD1-linked experimental model, the toxic factor produced and released via the exosomes by astrocytes mediates motor neuron death. That these findings may be relevant in vivo is corroborated by the fact that astrocyte-derived exosomes have been recently reported in rat cerebrospinal fluid [139]. The toxicity of astrocytes isolated from postmortem tissue from ALS patients to motor neurons and the involvement of SOD1 shown by Haidet-Philips et al. [140] support the ALS model.

MS is a demyelinating disease featuring axonal injury [141]. An increasing number of publications reveal the involvement of exosomes in the pathogenesis of MS. Exosomes play a pathological role by propagation of inflammation mediating the disruption of the BBB. Microglia-derived exosomes carry interleukin-1β (IL-1β) and MHC II and, therefore, support the spread of neuroinflammation [142]. Inflammation mediates the BBB disruption via the release of cytokines (e.g. interferon-γ (IFN-γ), TNF-α, IL-1β) [143], as well as the release of metalloproteinase-containing exosomes from surrounding cells (e.g. astrocytes) [144]. Cytokines, such as IFN-γ, can change the architectural organisation of tight and adherens junctions between cerebral endothelial cells of BBB [145]. Cytokines such as TNF-α and IL-1β promote the disruption of BBB via expression of inducible nitric oxide synthase [146]. In addition, metalloproteinases, which get delivered via exosomes, disrupt the BBB by degrading tight junction and basal lamina proteins, thereby leading to BBB leakage [147]. An untightened BBB enables leukocytes to migrate into the CNS [148].

Exosomes not only directly influence the pathology of MS but also provide information regarding the developmental status of this disease. These attributes favoured them as a therapeutic target as well as a diagnostic tool [149]. As an example, plasma of MS patients in the exacerbation phase contained 2.85-fold more CD31 (endothelial marker involved in acute injury) in exosomes than healthy control patients. The CD51 (marker for chronic injury of endothelium) content detected in plasma exosomes stays elevated in both exacerbation and remission phases. These examples mention that exosomes isolated from MS patients can provide information regarding the disease state [142, 148].

Other studies suggest the application of exosomes as an agent to transport immune-suppressive components through the BBB. For instance, MSC-derived exosomes can decrease inflammation and stop the demyelination of axons. For MSCs, an immune-suppressive functionality and assistance in neuronal repair is observed. Previously, MSCs were proposed as a treatment for autoimmune diseases such as MS [150–152]. In recent times, it was proposed that the application of MSC-derived exosomes could mediate the same effects [153]. Along with using pure MSC-derived exosomes, serum-isolated exosomes containing miR-219 can also stop demyelination and the progress of MS in mice. As a matter of fact, one interesting and almost recent finding was the potential remyelination effect of MSC-derived exosomes. Based on the study of Pusic et al. [60, 154], the application of IFN-γ-stimulated DC-derived exosomes containing a high level of miR-219 results in an increased remyelination of lysolecithin-induced demyelinated brain slice cultures. As supported by other studies, miR-219 has a major impact in differentiation of oligodendritic precursor cells and improvement of remyelination [154, 155]. In a later study, Pusic et al. [155] also showed that the application of serum-isolated exosomes from young rats improve remyelination in old ones. This could not be observed by using serum-isolated exosomes from old rats. However, the application of serum-isolated exosomes of environmental enriched (volitionally increased intellectual, social and physical activity) old rats improved the remyelination in old animals. In contrast to the exosomes derived from the old rats, both the exosomes from the young rats and the exosomes from the old environmental enriched rats contain miR-219, which is required for the production of myelinating oligodendrocytes [155]. In that study, it was also shown that the nasal delivery of the serum-isolated exosomes from young rats resulted in remyelination and, therefore, provided evidence for the potential therapeutic effects in the treatment of MS patients [59, 155].

For several CNS cell types, it was observed that exosomes play a crucial role in many biological processes. In addition, exosomes were studied concerning their role in several neurodegenerative and neurological diseases. These examinations offer promising key approaches towards diagnoses and treatments of this group of diseases. In this context, the ability of the exosomes to pass the BBB is an essential feature.

## Exosomes as a Tool for Diagnosis and Therapy

Exosomes are considered as a breakthrough in the diagnosis and as a potential therapeutic delivery system. They are suggested for diagnostic applications as they are isolable from all biological fluids and, therefore, provide a non/least invasive diagnostic method [156]. The majority of miRNA detectable in serum and saliva is concentrated in exosomes [157], where they are protected against RNases [158]. RNA and protein profiles of circulating exosomes can be correlated to specific diseases. As an example, serum-isolated exosomes from prostate cancer patients are selectively enriched with miR-141 in comparison to healthy controls [158]. Such diagnostic applications of exosomes were also described for colorectal cancer [159], ovarian cancer [160] and melanoma [161].

The diagnostic application of exosomes is not confined to the detection of miRNA. It was observed that the protein profile of exosomes isolated from the urine of patients with bladder cancer is different from the normal controls [162]. The detection of epidermal growth factor receptor variant III protein (EGFRvIII), a variant III EGFR deletion mutant missing 267 amino acids, plays a key role in gliablastoma diagnosis. Conventionally, biopsies are removed from the brain to check for EGFRvIII protein. The extraction of a biopsy from the brain is a complex invasive surgery. The same results can be obtained in a simple, much less invasive manner by the exosome screening for EGFRvIII protein, which is present in exosomes isolated from these patients. Therefore, their use can be a replacement for biopsy and, accordingly, extensively beneficial to the patients [163, 164].

Taken together, RNA and protein isolated from exosomes of cancer patients start to be used as diagnostic tools. Furthermore, analysis of exosomes makes it possible to categorise patients into different tumour risk classes enabling a more proper treatment towards the malignancy of tumours [165].

However, there are also some points that should be considered before relying thoroughly on the data obtained from exosomes in terms of diagnosis.

- Exosomes from different cell origins potentially have different compositions in their membrane, which affect their susceptibility to lysis. Different degrees of efficacy in lysis and isolation of the containing material could result in an alternative and probably false assumption of the result [166].
- Although up to now many studies have reported the high accurate association of some biomarkers with specific diseases in a large number of patients, the results of these studies do not match or, in some cases, even do not overlap. This discrepancy can be explained by several facts: (i) The most often used technique for diagnostic and assessing the nucleic acid content of exosomes is microarray technology; published data are often incomplete or incompletely annotated and therefore hard to reproduce [167]. (ii) As already mentioned, there are varieties of isolation techniques that can greatly influence the relative amount of the specific exosome cargo.
- In addition, the technology is rapidly evolving, but there is not yet a clear-cut unity for the analysis of large-scale profiles of small RNAs in exosomes [166].
- One last point is the probable medication. The source of exosomes is mainly from patients who have been most probably on medication before exosome isolation. The medication can alter some pathways and affect the molecules of interest.

A biomarker for clinical setting should be sensitive and specific for any individual case with a clear-cut definition. As it is not always possible to compare the patient with a specific control group, more studies and precise analyses (e.g. by next generation sequencing) are necessary to make exosomes routinely approachable for diagnosis means.

Exosomes are also suggested as a therapeutic delivery system. By their homing characteristic, they can deliver their cargo to specific targets over a long distance. Exosomes can also be used to deliver interfering RNA (siRNA) or pharmaceutically active substances [23, 168, 169]. It was shown that exosomes can be loaded with a specific cargo such as interference RNA (iRNA) and subsequently injected intravenously into mice. These injected exosomes were detected just in the target cells and no non-specific uptake was reported [168]. In the meantime, several groups could prevent tumour development—or suppress inflammatory responses in autoimmune patients—by using different sources of exosomes such as plasmacytoma cell-derived exosomes or exosomes derived from IL-10-treated DCs [156, 170]. In addition, it was shown that mouse exosome containing mRNA can be taken up by human cells, which consequently express the mouse proteins [10]. This observation potentially opens up a wide range of therapeutic aspects, such as treatment by transporting a specific protein to recipient cells lacking this specific protein, or a protein with a specific regulatory function. In addition to proteins, the potential transport of molecules could also include, e.g. mRNAs, miRNAs, iRNAs and drugs.

Overall, an exosome-based delivery system has particular benefits such as (i) specificity, as the exosomes deliver their cargo to a specific target; (ii) safety, as self-derived exosomes promote no undesired immunogenicity; and (iii) stability, not only the exosomes itself as nanostructures circulating in the blood were reported stable but also the content of exosomes are protected from RNases and proteases and, therefore, can be delivered in an intact form to the target cell [23]. Despite these benefits, there are some, until now, unsolved problems such as identifying and purifying a single subpopulation of endogenous cell-specific exosomes. Also, some findings are disservices to the clinical application of the isolated exosome:

- Exosomes possibly play a major role in the replication and propagation of transmissible pathogens [156]. This means that exosomes, which are derived from bacteria- or virus-infected cells, may contain factors such as pathogen-derived antigens or cytokines that activate a pro-inflammatory pathway. Pathogens also release vesicles that fuse with the plasma membrane of their target cells and release their contents into the cytosol of the recipient cell [8]. In addition, it is known that HIV uses the exosome pathway for its assembly and release [171]. Also, prion proteins mediate their intercellular transfer via exosomes [172].
- Exosomes have a diverse effect on health and diseases that are not thoroughly understood and controllable. Even though some exosomes can prevent tumour development, others provide a communication system between tumour cells and the surrounding tissues. In fact, exosomes are suggested as a nanodelivery system, which functions for tumour-associated proteins and, consequently, Cav-1-bearing exosomes. It is suggested that exosomes transfer Cav-1 to less aggressive cells and promote their invasive activity [173].

Considering these points, it was suggested to use exosome mimetic structures as an artificial treatment. Exosome mimetic delivery systems would be more controllable and scalable for clinical settings, and they can deliver anti-tumour drugs to the target cells [23]. Liposomes with a bilayer phospholipid and size of almost 100 nm can carry a variety of proteins and nucleic acids as well as pharmaceutically active substances. Such exosome mimetic structures also need specific targeting molecules. Thus, more information regarding targeting and releasing of exosomes is needed [174]. Currently, liposomal transport of drug molecules is under clinical trial [175], and lately, Gomez-Cabrero et al. developed specifically targeted liposomal nanoparticles and safely delivered doxorubicin (a chemotherapeutic drug commonly used to treat breast cancer) to a tumour microenvironment [176].

## Conclusion

Exosomes are small vesicles, which are formed by inward budding of MVBs and released via the plasma membrane. They are secreted by most if not all cells and can easily be isolated from biological fluids. These bilayer phospholipids can deliver mRNAs, miRNAs, proteins and, in some cases, DNAs. It was observed that the contents of exosomes are not entirely coincidently packed, but a selective process that conducts this loading. Due to normal biological processes in the cell, exosomal transport systems help cells to regulate other cells in distance and communicate with them. Cancer cells and malignant cells use this system to promote their invasive activity and suppress the immune system to act upon them.

Exosomes are considered a great diagnostic tool as they can be detected in biological fluids, they are very stable and their contents are protected. In addition, they are considered in many clinical studies as a delivery system to transport RNA, protein and drug molecules, especially as they can pass the BBB.

Nonetheless, there are also some not yet overcome disadvantages in using them for clinical terms. Their role is not completely understood, and in some cases, they can even activate pro-inflammatory pathways or suppress immune reactions. Thus, the use of exosome mimetic structures is favoured to target recipient cells and deliver pharmaceutically active substances. For developing such structures, a lot of topics stay open and need to be explored more in detail: (i) precise understanding of exosome biogenesis and targeting, (ii) providing a reliable method to target the exosomes in vivo and in vitro, *(*iii) isolation of a pure class of extracellular vesicles, (iv) clarification of the regulatory function of exosomes and (v) the effect of introducing excessive amounts of exosomes in an in vivo system. Besides, by improvement of the isolation techniques and detection of exosomes, we may rely more on these structures. Primary studies have also reported the successful delivery of chemotherapeutic drug-loaded exosome mimetic microvesicles to tumour tissue in vivo and in vitro [177].

Altogether, exosomes appear to be a specific and stable intercellular communication system, which can be used to obtain information more comfortably and less aggressively from inner organs and systems. Also, exosome mimetic structures can be designed to securely and specifically control the interactions between cells. For these aims, there is a lot to investigate regarding this fascinating delivery and target system.
